# The role of serotonin in personality inference: tryptophan depletion impairs the identification of neuroticism in the face

**DOI:** 10.1007/s00213-017-4619-4

**Published:** 2017-05-09

**Authors:** Robert Ward, Shubha Sreenivas, Judi Read, Kate E. A. Saunders, Robert D. Rogers

**Affiliations:** 1 School of Psychology, Bangor University, Brigantia Building, Penrallt Road, LL57 2AS Bangor, UK; 2 Department of Psychiatry, University of Oxford, Warneford Hospital, OX3 7JX Oxford, UK

**Keywords:** Serotonin, Personality, Neuroticism, Psychological distress

## Abstract

Serotonergic mechanisms mediate the expression of personality traits (such as impulsivity, aggression and anxiety) that are linked to vulnerability to psychological illnesses, and modulate the identification of emotional expressions in the face as well as learning about broader classes of appetitive and aversive signals. Faces with neutral expressions signal a variety of socially relevant information, such that inferences about the big five personality traits, including Neuroticism, Extraversion and Agreeableness, can be accurately made on the basis of emotionally neutral facial photographs. Given the close link between Neuroticism and psychological distress, we investigated the effects of diminished central serotonin activity (achieved by tryptophan depletion) upon the accuracy of 52 healthy (non-clinical) adults’ discriminations of personality from facial characteristics. All participants were able to discriminate reliably four of the big five traits. However, the tryptophan-depleted participants were specifically less accurate in discriminating Neuroticism than the matched non-depleted participants. These data suggest that central serotonin activity modulates the identification of not only negative facial emotional expression but also a broader class of signals about personality characteristics linked to psychological distress.

## Introduction

Serotonin mechanisms mediate the expression of personality traits linked to mental illnesses (Takano et al. 2007). High levels of Neuroticism have been associated with increased 5-HTT binding within the thalamus (Takano et al. 2007), while a number of characteristic emotional experiences associated with Neuroticism, such as anxiety, depression, hopelessness, somatization, guilt, hostility and affective temperament, have been linked to the 5-HTTPLR polymorphism of the serotonin transporter gene (Gonda et al. 2009). However, to date, there have been no systematic investigations of the role of serotonin activity in the identification of Neuroticism and other aspects of personality in other people.

Other evidence attests to the importance of serotoninergic mechanisms in the recognition of facial expressions of emotion including, but not limited to, fearful states (Harmer et al. 2003). Tryptophan depletion—producing temporary reductions in central serotonin activity (Moore et al. 2000)—impairs the accuracy of fear recognition in (non-clinical) healthy females and slows latencies for the recognition of fearful expressions in both healthy male and female adults (Harmer et al. 2003). By contrast, single doses of the selective serotonin reuptake inhibitor (SSRI), citalopram, improve fear recognition in healthy adults (Browning et al. 2007) and normalize recognition of fearful (and positive) facial expressions in previously depressed individuals (Bhagwagar et al. 2004). These effects are likely to be mediated by altered signaling within neural circuitry encompassing the amygdala (Harmer et al. 2004), suggesting that anti-depressant modulation of monoaminergic activity within limbic circuits alters sensitivity to facial emotional signals (as well as broader cognitive biases) to support delayed therapeutic effects (Anderson et al. 2011; Booij and Van der Does 2011; Hayward et al. 2005; Harmer et al. 2006; Walsh and Harmer 2015). However, beyond emotional expressions, the face transmits a variety of other informative cues to social traits. In this study, we examined whether serotoninergic neuromodulation influences the accuracy of personality inferences, as cued by neutral facial expressions.

Research on trait inferences shows that observers can identify personality and other important social traits, with modest levels of accuracy, using only impoverished cues. For example, Borkenau and Liebler (1992) established that, even at ‘zero-acquaintance’, observers could draw accurate personality inferences from short video clips of an individual walking into a room, or quietly facing the camera. Such nonverbal ‘thin slices’ of behaviour can be famously diagnostic of consensual longer-term impressions; for example, teachers’ end-of-semester ratings can be predicted from judges’ reactions to a few seconds of silent video clips of those teachers (Ambady and Rosenthal 1992). While accurate trait inferences can be drawn on the basis of very short exposures (Borkenau et al. 2009; Rule et al. 2009), accuracy is also affected by the quantity and quality of cues available (Funder and Colvin 1988; Carney et al. 2007). In particular, the availability of other cues under individuals’ control—such as their hairstyles, clothing, cosmetics and facial expressions—can improve accuracy of trait judgements (Naumann et al. 2009).

However, even when controllable cues are minimal, facial appearance under constrained and standardized conditions (such as in a passport photograph) can drive accurate trait inferences. Emotionally neutral facial appearances, without evident clothing, cosmetics, or hairstyle cues, can allow accurate judgements of socially relevant traits as diverse as trustworthiness (Stirrat and Perrett 2010), women’s reproductive success (Pflüger et al. 2012) and even clinically diagnosed borderline personality disorder (Daros et al. 2016). The accuracy of trait inferences based upon neutral faces suggests an important, but under-explored, channel of social communication in which individuals continuously and involuntarily broadcast cues about behavioural predispositions to observers and potential social partners.

Of primary interest for the present study are findings that factor-analytic personality traits, and in particular trait Neuroticism, can be identified from composite images of neutral facial appearance (e.g. Little and Perrett 2007; Kramer and Ward 2010; Jones et al. 2012). Neuroticism is marked by a tendency towards fearful emotions including anxiety, depression, fear and worry (Vinberg et al. 2014; McWilliams 2003). Neurotic traits are also related to the frequency and intensity of negative emotions (Verduyn and Brans 2012). Of all the big five personality traits, Neuroticism would be the most closely related to fear (Nettle 2006). Given that serotonin plays an important role in the recognition of emotional expressions and, in particular, fearful states (e.g. Harmer et al. 2003), we hypothesised that serotonin activity plays a role in identifying trait Neuroticism from the face. We therefore investigated, for the first time, the role of serotonergic activity in making accurate personality discriminations from facial cues. We tested the specific prediction that tryptophan depletion impairs accurate identification of Neuroticism from neutral facial expressions in healthy adult volunteers.

## Method

The experiment was approved by a National Health Service (England) Research Ethics Committee. All participants provided written, informed consent.

### Participants

Fifty-three males and females were recruited from the student population of Oxford University and the local community. One female was excluded on the basis of 0% accuracy in discriminating Neuroticism (3.7 SDs from her group mean). No other participant had 0% accuracy. Participants were assessed by an experienced psychiatrist against explicit exclusion criteria using the Structured Clinical Interview for DSM-IV-TR Axis I Disorders (First et al. 2002).

Exclusion criteria included (i) the presence or history of serious physical illness, (ii) history of neurological disorder or head injury, (iii) current, previous or family history of mood-related illness (unipolar or bipolar disorder), (iv) current or previous substance misuse or dependence, (v) other significant psychiatric illness, (vi) any illness or indicators that preclude blood-sampling and (vii) pregnancy or breast-feeding.

### Design

The study consisted of a between-subjects, double-blind design. Twenty-five participants (10 men, 15 women) were randomly selected to consume an amino-acid drink without *l*-tryptophan (T− treatment), and 27 participants (16 men, 11 women) were randomly selected to consume an amino-acid drink with *l*-tryptophan (T+ treatment).

### Materials

#### Amino acids

Amino acids were supplied by Nutricia or Cambridge Bioscience. Amounts for males and females, respectively, were the following: *l*-alanine (5.5 g; 4.58 g), *l*-arganine (4.9 g; 4.08 g), *l*-cystine (2.7 g; 2.25 g), glycine (3.2 g; 2.67 g), *l*-isoleucine (8.0 g; 6.67 g), *l*-leucine (13.5 g; 11.25 g), *l*-lysine monohydrochloride (11.0 g; 9.17 g), *l*-methionine (3.0 g; 2.5 g), histidine (3.2 g; 2.67 g); *l*-phenylalinine (5.7 g;4.75 g), *l*-proline (12.2 g; 10.17 g), *l*-serine (6.9 g; 5.75 g), *l*-threonine (6.5 g; 5.42 g), *l*-tyrosine (6.9 g; 5.75 g) and *l*-valine (8.9 g; 7.42 g). The T+ drink contained *l*-tryptophan (2.3 g; 1.92 g). Tastes were masked with 5 g (15 cals; 1.3 g carb.) of citric (or malic) acid (cherry-and-vanilla or grapefruit) and artificial sweetener.

#### Psychometric assessments

Following the screening interview, participants completed the (i) trait and state Positive and Negative Affect Scales (Watson et al. 1988), (ii) Beck’s Depression Inventory (BDI-II) (Beck et al. 1996), and (iii) Buss-Perry Aggression Questionnaire (Buss and Perry 1992). The PANAS consists of two subscales of 20 items, rated using a 5-point Likert scale, to measure positive and negative aspects of emotional experience. Cronbach’s α coefficients for the state (i.e. momentary) version of the positive affect and negative affect subscales have been reported as .90 and .87, respectively (Watson et al. 1988).

The BDI-II consists of 21 statements that capture the frequency of depressive symptoms over the previous 14 days, with total scores tested against cut-offs to indicate minimal to severe depression (Beck et al. 1996). It has strong convergent and criterion reliability. A Cronbach’s α coefficient of 0.94 indicates high internal consistency (Arnau et al. 2001). Finally, our participants completed the Standard Progressive Raven’s Standard Progressive Matrices to assess non-verbal cognitive ability (Raven et al. 2004).

### Personality trait discrimination task

The trait discrimination task used composites of female faces, reflecting the correlated facial appearance of women who share similar levels of personality traits (e.g. Kramer and Ward 2010; Jones et al. 2012). We used the full-face stimulus set created by Kramer and Ward (2010), which was made by averaging neutral face photos from women scoring highest and lowest on self-report measures of each big five personality traits (Agreeableness, Openness, Conscientiousness, Extraversion and Neuroticism), as measured on the Mini International Personality Item Pool (Mini-IPIP, Donnellan et al. 2006). Thus, the composite face-pairs reflected regularities in facial appearance which are correlated with specific personality traits. In addition, the stimuli set included two composites which were not based upon the Big Five traits: physical health (used by Kramer and Ward 2010) and depressive symptoms (as described by Scott et al. 2013). These two additional composites were presented only once in each stimulus set, providing insufficiently reliable patterns of discriminations; they are not discussed further.

**Fig. 1:**
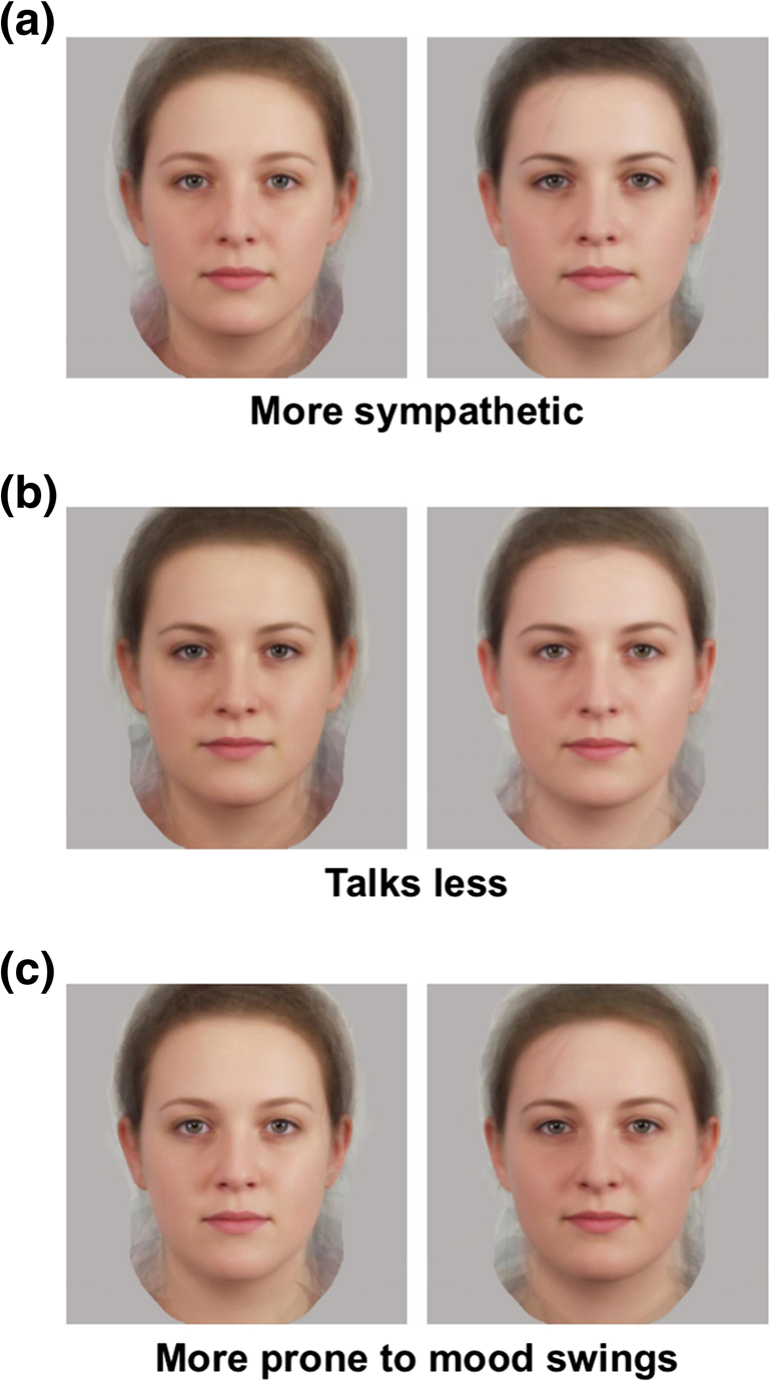
Face stimuli and example discrimination statements for **a** Agreeableness, **b** Extraversion and **c** Neuroticism. In each case, the face on the *left* is a composite of 15 women scoring highest on the trait measure, and the face on the *right* a composite of 15 women scoring lowest on that measure (see Kramer and Ward 2010 for details). Participants were asked to identify which of the two faces best matched the accompanying discrimination statement

On each presentation (hereafter, trial), one high and one low composite for a personality trait were presented, randomly positioned on either side of centre on a standard computer display, along with a discrimination statement relevant to that trait (e.g. ‘More prone to mood swings’ for trait Neuroticism, see Fig. 1). Participants made an unspeeded mouse-click on the face best matching the discrimination statement. Each trait was presented four times, using four different discrimination statements (items) adapted directly from the Mini-IPIP items (Donnellan et al. 2006).

## Procedure

All participants followed a low-protein diet for 24 h before ingesting an amino-acid drink on the morning of the experiment. On arrival at the laboratory, participants completed baseline ratings of state positive and negative affect using the PANAS (Watson et al. 1988). Blood samples (6 ml) were taken for baseline measurements of plasma tryptophan levels and tryptophan/large-neutral-amino-acid (LNAA) ratios. Participants then ingested their allocated amino acid drinks. Five hours later (+5 h), participants provided follow-up ratings of state affect with the PANAS, as well as a second blood sample. Participants then completed cognitive assessments and social exchange games that included the computerized trait discrimination task reported here.

### Data analysis

Differences in the number of males and female participants in the T− and the T+ groups were tested with a *χ* ^2^ test. Group matching of age, cognitive ability (scored with the Raven’s Standard Progressive Matrices; Raven et al. 2004), trait affect (scored with the Positive and Negative Affect Scale; Watson et al. 1988) and recent depressive symptoms (scored with the Beck Depression Inventory II; Beck et al. 1996) were tested with two-sample *t* tests. Changes in total plasma tryptophan, the ratio of plasma tryptophan to LNAAs, and state positive and negative affect (as measured by the PANAS) were assessed by separate repeated measures ANOVAs with the single between-subject factor of treatment (T− vs T+) and the within-subject factor of time (baseline vs +5 h).

Using the same full-face stimuli as here, Kramer and Ward (2010) demonstrated that healthy young adults (comparable to our participants) are able to discriminate reliably Neuroticism, Extraversion and Agreeableness but not Openness or Conscientiousness. Similar accuracy levels of trait discrimination are reported by Kramer and Ward (2011) using hemi-face versions of these stimuli (that is, presenting just the left or right half of the stimuli). Therefore, we focused our analyses upon the most robustly discriminated traits of Neuroticism, Extraversion, and Agreeableness. First, we used one-sample *t* tests to assess all participants’ discrimination accuracy against chance (0.5) and two-sample *t* tests to test the prediction that T− participants were less accurate in discriminating Neuroticism and, perhaps, other traits compared to T+ participants. Fifty-two participants provide a statistical power of approximately 0.8 at *α* = .05 (one-sided) to detect medium effect sizes (0.5) in the one-sample *t* tests and medium-to-large effect sizes (0.7) in the two-sample tests.

Since this is the first study of tryptophan depletion and the discrimination of personality traits from the face, we also used a mixed-effects binomial model (Bates 2010) to test whether the inclusion of the above variables in the model moderated the reliability of treatment effects. The model included random intercept terms for participant and for item, and a random slope for treatment by item, and fixed effects for the remaining predictors, comprising tryptophan depletion, and the demographic and psychometric variables above. All non-categorical predictors (i.e. other than sex and treatment) were zero-centred and scaled to variance of 1.

## Results

The demographic and psychometric characteristics of the T− and T+ participants are shown in Table 1. As per standard practice (Rogers et al. 2003; Bilderbeck et al. 2014), the groups were closely matched for age, cognitive ability (as measured by the Raven’s Progressive Matrices (Raven et al. 2004)), recent depressive symptoms (as measured by the BDI (Beck et al. 1996)), trait positive and negative affect (as measured with the trait version of the PANAS (Watson et al. 1988)) and trait aggression (Buss and Perry 1992), all *t*(50) < 2.0.

**Table 1:** Demographic and psychometric characteristics, plus plasma tryptophan measurements, of 25 healthy adults who completed a tryptophan depletion protocol and consumed an amino acid drink without tryptophan (T− participants) and 27 healthy adults who consumed an amino acid drink that did contain tryptophan (T+ participants)

	T− participants	T+ participants
Sex (male/female)	10:15	16:11
Age	25.2 (1.21)	24.1 (1.20)
Depressive symptoms (BDI)	0.68 (0.34)	1.30 (0.30)
Trait +ve affect (PANAS)	37.5 (1.38)	35.9 (1.15)
Trait −ve affect (PANAS)	11.4 (0.39)	12.9 (0.83)
Total Aggression (Buss-Perry)	44.4 (2.16)	50.3 (2.18)
Raven’s Matrices	53.4 (0.69)	54.4 (0.76)
Plasma trypt. (μg/ml) 0 h	10.2 (0.39)	10.4 (0.38)
Plasma trypt. (μg/ml) +5 h	4.0 (0.68)	21.2 (1.11)
Tryptophan LNAA ratio 0 h	0.14 (0.01)	0.14 (0.01)
Tryptophan LNAA ratio + 5 h	0.03 (0.01)	0.16 (0.01)
State +ve affect (PANAS) +0 h	28.2 (1.66)	29.0 (1.41)
State +ve affect (PANAS) +5 h	27.2 (1.58)	29.0 (1.59)
State −ve affect (PANAS) +0 h	12.2 (0.57)	12.6 (0.45)
State −ve affect (PANAS) +5 h	11.5 (0.58)	11.1 (0.27)

Beck’s Depression Inventory (BDI-II; Beck et al. 1996), trait and state Positive and Negative Affect Schedule (PANAS; Watson et al. 1988), Raven’s Matrices (Raven et al. 2004) and Aggression Questionnaire (Buss and Perry 1992)

As expected (Moore et al. 2000), plasma total tryptophan concentrations showed significant divergence between baseline and +5 h in the T− and T+ participants (see Table 1), *F*(1,45) = 170.1, *p* < .00001. Specifically, total tryptophan decreased in the T− participants from 10.2 to 4.0 μg/ml, *t*(22) = 7.20, *p* < .00001, but increased in the T+ participants from 10.4 to 21.2 μg/ml, *t*(23) = 11.09, *p* < .00001 (note: 3 T+ and 2 T− participants were excluded from these tests because of missing blood samples in the morning or afternoon). The ratio of plasma tryptophan to other LNAAs also showed different changes following the T− and T+ drinks (see Table 1), *F*(1, 45) = 69.6, *p* < .00001. Here, however, only the tryptophan/LNAA ratios following the T− treatment were reduced from .14 to .03, *t*(22) = 10.4, *p* < .00001, but the change from .14 to .16 following the T+ treatment was not significant, *t*(23) = 1.28, *p* = .215. At +5 h, both plasma total tryptophan and the tryptophan/LNAA ratios were reduced in the T− participants compared to the T+ participants, *t*(45) = 12.4, *p* < .00001, and *t*(45) = 11.0, *p* < .00001, respectively.

Overall, participants’ state negative affect (as scored by the PANAS; Watson et al. 1988) showed a significant decline between baseline and +5 h (see Table 1), *F*(1, 50) = 12.0, *p* < .002. State positive affect was unchanged, *F*(1, 50) < 1. Tryptophan depletion did not produce any marked differential changes in either state positive or negative affect (Table 1); all *F*s(1, 50) < 1.6, all *p*s > 0.2.

### Trait accuracy

Overall, and consistent with Kramer and Ward (2010), participants discriminated Neuroticism with significantly greater accuracy than chance by a one-sample *t* test, M = .70, SE = .035, t(51) = 5.7, *p* < .0001, *d* = .79. This was also true of Extraversion, M = .75, SE = .038, *t*(51) = 6.65, *p* < .0001, *d* = .92, and Agreeableness, M = .63, SE = .042, *t*(51) = 3.09, *p* = .003, *d* = .43. Like Kramer and Ward (2010), our participants were unable to discriminate Openness reliably, M = .50, SE = .037, *t*(51) = .13, *p* = .9, *d* = .02, but, unlike Kramer and Ward (2010), they were able to discriminate Conscientiousness, M = .60, SE = .036, *t*(51) = 2.8, *p* = .008, *d* = .38.

**Fig. 2:**
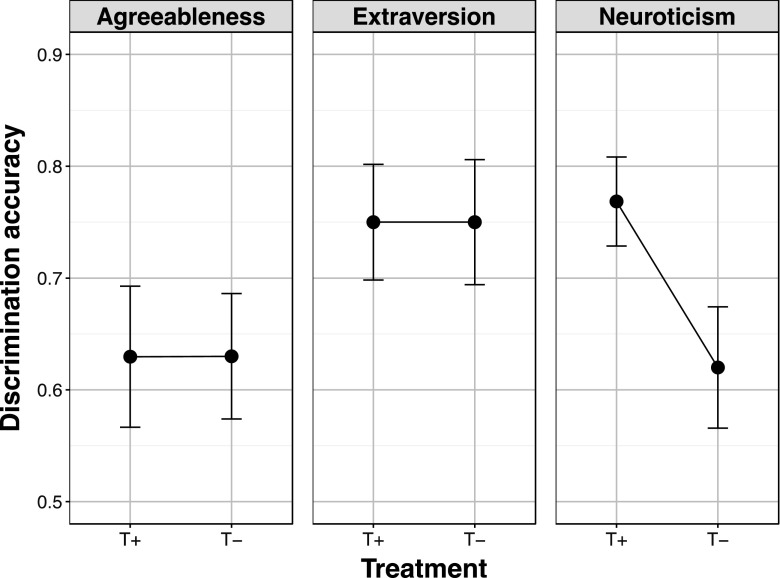
Mean proportionate accuracy on the two-alternative forced-choice trait discrimination of Agreeableness, Extraversion, and Neuroticism in 27 healthy participants who had consumed an amino acid drink that did contain tryptophan (T+ participants) and 25 healthy adult participants who had consumed an amino acid drink without tryptophan (T− participants). *Error bars* represent 1 standard error

Separate comparisons showed that the T− participants showed significantly poorer discrimination of Neuroticism compared to the T+ participants by a two-sample *t* test (see Fig. 2), *t*(50) = 2.23, *p* = .030, *d* = .62. By contrast, the discrimination accuracies of the T− participants showed no significant changes compared to the T+ participants for both Extraversion and Agreeableness (see Fig. 2) or indeed Openness and Conscientiousness (see Table 2), all *t*s(50) < 1, *p*s > .3, *d* < .26).

**Table 2:** Mean discrimination accuracy (together with standard errors) for Agreeableness, Extraversion, Neuroticism, Openness and Conscientiousness in 25 healthy adults who had completed a tryptophan depletion protocol and consumed an amino acid drink without tryptophan (T− participants) and 27 matched healthy adults who had consumed an amino acid drink that did contain tryptophan (T+ participants)

	T− participants	T+ participants
Agreeableness	0.63 (0.056)	0.63 (0.063)
Extraversion	0.75 (0.056)	0.75 (0.052)
Neuroticism	0.62 (0.054)	0.77 (0.040)
Conscientiousness	0.57 (0.059)	0.63 (0.045)
Openness	0.47 (0.056)	0.54 (0.048)

Discrimination accuracy for Neuroticism was examined in detail with a binomial mixed-effects model (Bates 2010) (see Table 3). Female participants showed lower accuracy than male participants, *β* = −0.796, SE = 0.360, *p* = 0.027, and the T− participants showed significantly lower accuracy in identifying neurotic faces, *β* = −0.934, SE = .358, *p* = 0.0091). No significant effect was found for other predictors (all *βs <* 0.33). The effect of treatment was numerically larger for women than men (T+: women = .75, SE = .058; men = .78, SE = .055; T−: women = .55, SE = .065, men = .725, SE = .087). However, an additional treatment × sex interaction term was not significant, *β* = 0.332, SE = 0.699, and did not improve the model, *χ* ^2^(1) = 0.21.

**Table 3:** Mixed-effects binomial modelling of discrimination on Neuroticism trials

(Intercept)	1.00 (.375)
Sex (male = 0; female = 1)	−.796 (.360)*
Age	.131 (.190)
Buss-Perry total	−.327 (.210)
BDI	.061 (.193)
Trait PANAS +ve	−.047 (.166)
Trait PANAS −ve	−.267 (.189)
Ravens	.004 (.178)
Treatment (T+ = 0; T− = 1)	−.934 (.358)**

Standardized weighting (standard error) for non-categorical factors

## Discussion

To our knowledge, this is the first demonstration that serotonin function supports the perceptual discrimination of personality. These findings show that tryptophan depletion—producing temporary reductions in central serotonin activity (Moore et al. 2000; Bilderbeck et al. 2014)—diminishes the capacity of healthy adults to discriminate Neuroticism, but not other traits, in other people on the basis of facial cues. The T− and the T+ participants were closely matched in terms of sex, age and cognitive ability. Consistent with previous studies involving healthy adult volunteers with no personal or family history of depression (Rogers et al. 2003; Bilderbeck et al. 2014; Wood et al. 2006), there were no marked differences between our T− participants and T+ participants in terms of their state positive or negative affect, either before or following consumption of the amino acid drinks.

Our findings expand our understanding of serotoninergic mechanisms in processing of emotional and social signals in other people. Substantial evidence attests to the monoaminergic modulation of emotional recognition in the face (Walsh and Harmer 2015) and the processing of other appetitive and aversive signals (Boureau and Dayan 2011; Crockett et al. 2012; Hindi et al. 2012). Neuroticism is strongly associated with fearfulness and vulnerability to anxiety, depression and negative rumination (Fergusson et al. 1989; Nettle 2006; Vinberg et al. 2014; Verduyn and Brans 2012). Thus, our finding that tryptophan depletion impairs the accuracy of Neuroticism discrimination suggests that serotonergic activity mediates the ability to discriminate subtle cues that signal vulnerability to psychological distress in the absence of expressed negative emotional states.

Serotonergic mechanisms influence the expression of social behaviours in both humans (Knutson et al. 1998; Moskowitz et al. 2001; Tse and Bond 2002; Young and Leyton 2002) and non-human primates (Raleigh et al. 1981; Raleigh et al. 1985) and, in particular, a wide variety of social exchanges with partners and group members (Colzato et al. 2013; Higley et al. 1996; Crockett et al. 2012; Crockett et al. 2008; Wood et al. 2006; Bilderbeck et al. 2014). Twelve days administration of tryptophan in healthy (non-clinical) adults decreases (perceived) quarrelsomeness and increases dominance (Moskowitz et al. 2001, 2003). The present findings suggest that serotonergic influences over social behaviours also moderates sensitivity to the facial signals of vulnerability to distress in (potential or actual) social partners. Important social behaviours—for example, the selection of mates and the pursuit of affiliative relationships—are influenced by the perception and then the appraisal of facial appearances (e.g. Rhodes 2006). The present data indicate that serotonergic activity mediates these behaviours by its contribution to perceptual sensitivity to cues of psychological vulnerability.

These results also raise the possibility that psychological disorders, such as depression, whose pathophysiology includes serotonergic dysfunction (Bhagwagar and Cowen 2008; Bhagwagar and Cowen 2008; Cowen 2008) are associated with mistaken inferences about other peoples’ personalities, especially around vulnerability to psychological distress and illness. Accurate discrimination of mental health can be accompanied by other negative and potentially incorrect inference. For example, Scott et al. (2013) and Daros et al. (2016, Study 2A) report high levels of accuracy in judging depressive symptoms from facial expressions, but importantly, the faces of individuals with poor mental health were mistakenly judged as lower in warmth and friendliness (Scott et al. 2013), and more negatively in their emotional expressions (Daros et al. 2016). To the extent that depressive symptoms are mediated by serotonergic dysfunction (Bhagwagar and Cowen 2008; Cowen 2008), there may be a cycle of psycho-pathogenic effects: (1) Healthy observers mistakenly appraise depressive individuals with negative characteristics (Coyne 1976); (2) serotonin dysfunction in depression triggers misidentification of personality of others. Together, these misperceptions may disrupt social relationships in depressed people.

Tryptophan depletion had no marked effects upon the discrimination of other big five traits shown to our participants (Agreeableness, Extraversion, Openness and Conscientiousness), suggesting that serotonin activity plays a particular role in the discrimination of Neuroticism. The specificity of the effects of tryptophan depletion reported here also rules out a single mechanism for personality discrimination such as, for example, the selection of the more attractive of the two composites presented as the more desirable characteristic (i.e. highly Agreeable, Extraverted, and Stable). Rather, our data suggest that, like the discrimination of emotional expressions (e.g. Calder and Young 2005), trait inference based upon facial characteristics depends upon dissociable mechanisms. Future investigation will need to delineate the extent to which discrimination of personality from the face share cognitive and neurochemical substrates with the discrimination of emotional expressions (c.f. serotonin and noradrenaline; Harmer et al. 2004).

The present study used the same stimuli and, broadly, replicates the findings of Kramer and Ward (2010) and, in particular, the accurate discrimination of full-face composites for the traits Agreeableness, Extraversion, and Neuroticism. However, in contrast to Kramer and Ward, our participants had completed a protocol that included prior dietary (protein) restriction, consumption of amino acid drinks and a full day of testing. Collectively, these two datasets, therefore, suggest some robustness in the accuracy of individuals’ discrimination of these stimuli. However, in contrast to Kramer and Ward (2010), our participants showed significantly better than chance discrimination of Conscientiousness. Therefore, at present, we cannot be certain about the consistency of Conscientiousness identification across study protocols or participant populations, and are reluctant to draw strong conclusions about the absence of changes in Conscientiousness discrimination following tryptophan depletion.

In these data, women were significantly less accurate than men in the discrimination of Neuroticism. This is somewhat unusual, as women are often found to be more sensitive than men in trait inference tasks, and are rarely found to be worse (e.g. Hall and Mast 2008). This outcome probably reflects the differential effects of the tryptophan depletion across sexes. Tryptophan depletions’ cognitive and emotional effects are often greater in women than men (Booij et al. 2002), including effects upon the recognition of fearful expressions (Harmer et al. 2003). Sex differences involving tryptophan depletion may reflect differences in hormonal activity or the distribution of receptors (Fehr et al. 2000; Zhang et al. 1999). Here, although not statistically significant, impaired discrimination of Neuroticism following tryptophan depletion was numerically more marked in the women than men. Therefore, the significant main effect of sex upon may reflect only the greater impact of depletion upon women’s accuracy for Neuroticism.

Finally, changes in the recognition of facial expressions of emotion following single treatments of serotonergic or noradrenergic anti-depressants (Bhagwagar et al. 2004; Browning et al. 2007) appear to form part of a complex of cognitive adjustments that presage the therapeutic effects upon mood following continued treatment in clinical populations (Walsh and Harmer 2015). Further experimentation with tryptophan loading protocols (Murphy et al. 2006, 2009), single or sub-chronic treatments with anti-depressants could indicate whether the accuracy of Neuroticism inferences from the face can be improved by *enhancing* serotonergic activity and other personality inferences in treated and untreated depressed patients. Similarly, trait inferences about potential social partners may regulate approach and withdrawal behaviours, highlighting the issue of how serotonergic modulation of these judgements relates to the activity of the inter-dependent peptide systems implicated in both mood and social bonding, such as oxytocin (Dolen 2015; Flight 2013).

Notwithstanding these future possibilities, these data provide preliminary evidence that transient reductions in serotonin activity, produced by tryptophan depletion, impair the discrimination between high versus low Neuroticism on the basis of facial cues. These findings suggest that serotonin activity modulates the detection of physical signals of vulnerability to depression and psychological distress.
